# Shedding of Viable Virus in Asymptomatic SARS-CoV-2 Carriers

**DOI:** 10.1128/mSphere.00019-21

**Published:** 2021-05-19

**Authors:** Takayuki Murata, Aki Sakurai, Masahiro Suzuki, Satoshi Komoto, Tomihiko Ide, Takuma Ishihara, Yohei Doi

**Affiliations:** a Department of Virology and Parasitology, Fujita Health University School of Medicine, Toyoake, Aichi, Japan; b Department of Infectious Diseases, Fujita Health University School of Medicine, Toyoake, Aichi, Japan; c Department of Microbiology, Fujita Health University School of Medicine, Toyoake, Aichi, Japan; d Center for Joint Research Facilities Support, Research Promotion and Support Headquarters, Fujita Health University School of Medicine, Toyoake, Aichi, Japan; e Innovative and Clinical Research Promotion Center, Gifu University Hospital, Yanagido, Gifu, Japan; University of Pittsburgh School of Medicine

**Keywords:** COVID-19, COVID-19 nucleic acid testing, SARS-CoV-2, asymptomatic infections, carrier state, cell culture techniques, cytopathogenic effect, infectivity, virus shedding, whole-genome sequencing

## Abstract

Information regarding the infectivity of severe acute respiratory syndrome coronavirus 2 (SARS-CoV-2) in asymptomatic carriers is scarce. In order to determine the duration of infectivity and its correlation with reverse transcription-PCR (RT-PCR) results and time since initial positive PCR test in this population, we evaluated SARS-CoV-2 cell infectivity in nasopharyngeal samples longitudinally obtained from asymptomatic carriers who disembarked from a cruise ship during a COVID-19 outbreak. Of 166 nasopharyngeal samples collected from 39 asymptomatic carriers every 48 h until two consecutive negative PCR test results were obtained, SARS-CoV-2 was successfully isolated from 9 PCR-positive samples which were obtained from 7 persons (18%; 7/39). Viable viruses were isolated predominantly within 7 days after the initial positive PCR test, except for one person who shed viable virus until day 15. The median crossing point (Cp) value of RT-PCR of culture-positive samples was 24.6 (interquartile range [IQR], 20.4 to 25.8; range, 17.9 to 30.3), and Cp values were significantly associated with isolation of viable virus (odds ratio, 0.496; 95% confidence interval [CI], 0.329 to 0.747; *P* value, 0.001), which was consistent with existing data for symptomatic patients. Genome sequence analysis of SARS-CoV-2 samples consecutively obtained from a person who shed viable virus for 15 days identified the emergence of two novel single nucleotide variants (C8626T transition and C18452T transition) in the sample collected on day 15, with the latter corresponding to an amino acid substitution in nonstructural protein 14. The impact of these mutations on prolonged viable-virus shedding is unclear. These findings underscore the potential role of asymptomatic carriers in transmission.

**IMPORTANCE** A growing number of studies suggest the potential role of asymptomatic SARS-CoV-2 carriers as a major driver of the COVID-19 pandemic; however, virological assessment of asymptomatic infection has largely been limited to reverse transcription-PCR (RT-PCR), which can be persistently positive without necessarily indicating the presence of viable virus (e.g., replication-competent virus). Here, we evaluated the infectivity of asymptomatic SARS-CoV-2 carriers by detecting SARS-CoV-2-induced cytopathic effects on Vero cells using longitudinally obtained nasopharyngeal samples from asymptomatic carriers. We show that asymptomatic carriers can shed viable virus until 7 days after the initial positive PCR test, with one outlier shedding until day 15. The crossing point (Cp) value of RT-PCR was the leading predictive factor for virus viability. These findings provide additional insights into the role of asymptomatic carriers as a source of transmission and highlight the importance of universal source control measures, along with isolation policy for asymptomatic carriers.

## INTRODUCTION

Since the early phase of the coronavirus disease 2019 (COVID-19) pandemic, asymptomatic carriers of severe acute respiratory syndrome coronavirus 2 (SARS-CoV-2), who were infected with SARS-CoV-2 but never developed symptoms, have been identified through contact tracing and surveillance. Despite a growing number of studies suggesting the potential role of asymptomatic carries as a major driver of transmission (1, 2), information regarding the infectivity of asymptomatic SARS-CoV-2 carriers is limited.

Reverse transcription PCR (RT-PCR) has been the gold standard for COVID-19 diagnostic testing and screening. RT-PCR testing results, however, can be persistently positive without necessarily indicating the presence of viable virus (e.g., infectious virus or replication-competent virus) and transmissibility to others. Recent studies using viral culture as a surrogate marker for infectivity have shown that viable virus is not recovered after 10 days following symptom onset in mild to moderate COVID-19 (3–5). Among patients with severe COVID-19, the median duration of viable virus shedding was 8 days (interquartile range [IQR], 5 to 11 days) after onset, and the probability of detecting viable virus dropped to below 5% 15 days after onset of symptoms (6). To date, detection of viable virus has been documented in asymptomatic patients (5), but the kinetics of viable virus shedding among asymptomatic patients, who make up 16 to 45% of all infected patients, are not known (7, 8). Here, we investigated SARS-CoV-2 cell infectivity in nasopharyngeal specimens obtained from asymptomatic persons who contracted the virus during the COVID-19 outbreak on the cruise ship Diamond Princess using cell culture and correlated it with crossing point (Cp) values of RT-PCR.

## RESULTS

**FIG 1:**
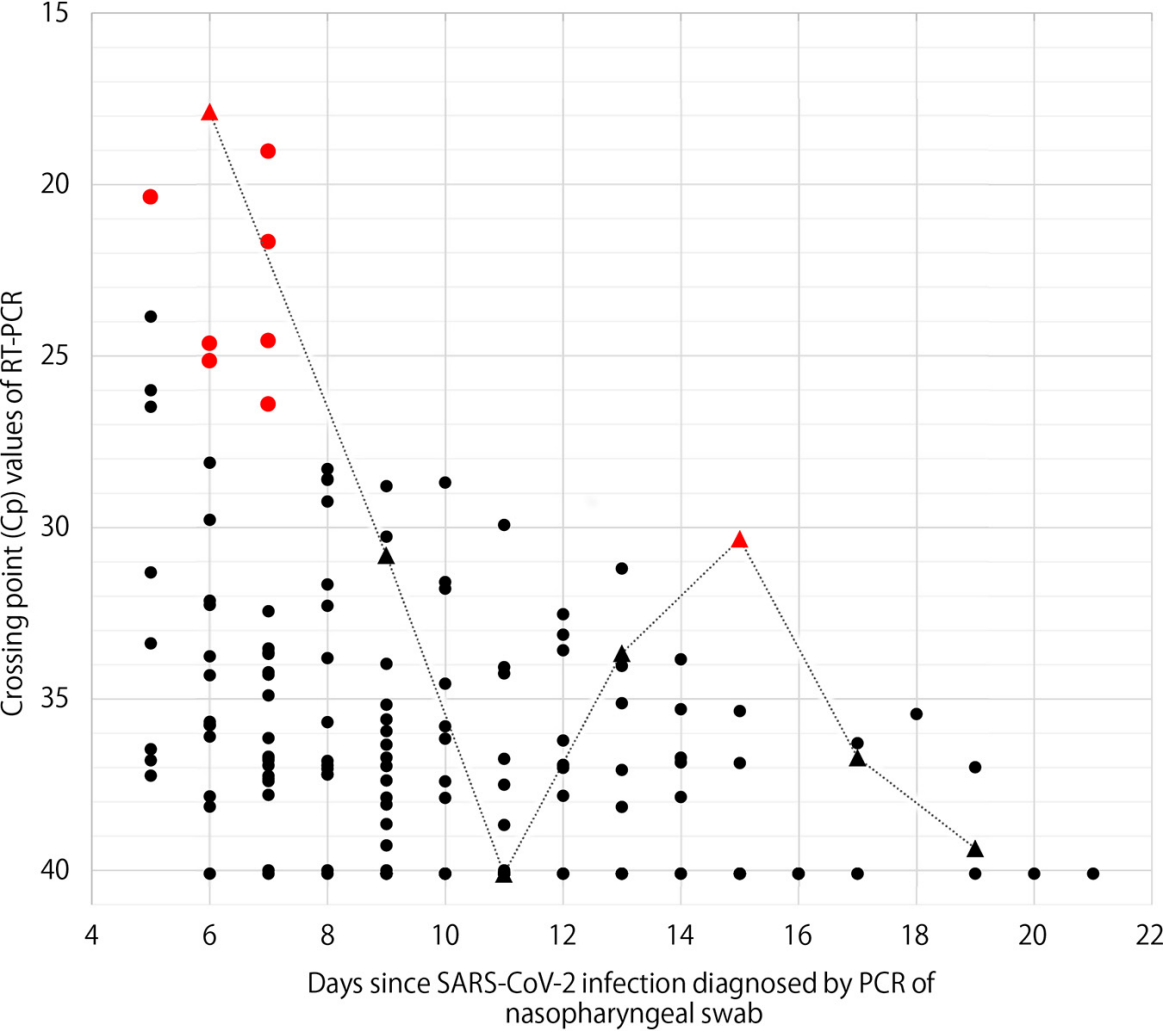
Crossing point (Cp) values of RT-PCR and result of cell culture in asymptomatic SARS-CoV-2 carriers, according to number of days since SARS-CoV-2 infection diagnosis by PCR of nasopharyngeal swabs. Red dots indicate samples in which cytopathic effect (CPE) was observed, and black dots represent culture-negative samples. Triangles connected with a dotted line correspond to samples obtained from Carrier_1. With fluorescence-based real-time PCR, the number of cycles at which fluorescence signal from amplification exceeds the background fluorescence level is determined as the crossing point, threshold cycle (*C_T_*), or other values by different instrument manufacturers. While there are differences in how they are calculated, they are considered biologically equivalent in that a lower value correlates with a higher copy number of the target nucleotide sequence.

Of 90 asymptomatic SARS-CoV-2 carriers, 39 had more than two consecutive or nonconsecutive positive PCR test results at the hospital. The median age was 67 (IQR, 57 to 72; range, 9 to 77) and 54% (21/39) were female. Fourteen (36%) had coexisting medical conditions, such as hypertension and diabetes. The first PCR test at the hospital took place after a mean of 6 days following the initial positive PCR test on the ship. A median of 3 samples (IQR, 2 to 4; range, 2 to 7) were obtained from each person until two consecutive negative PCR test results were obtained. Eventually, a total of 166 samples were analyzed using cell cultures, consisting of 116 PCR-positive samples and 50 PCR-negative samples. The latter were obtained between PCR-positive samples (*n* = 11) or as the first of two consecutive negative PCR tests (*n* = 39). SARS-CoV-2 was successfully cultured from 9 (7.8%) of PCR-positive samples, whereas none of the PCR-negative samples showed cytopathic effect (CPE). Samples with positive culture were obtained from 7 carriers with a median age of 71 (IQR, 66 to 74; range, 60 to 77), 5 of whom were female. The last positive CPE was observed in samples collected a median of 7 days (IQR, 6 to 7; range, 6 to 15) after the initial positive PCR test (day 0) (Fig. 1; also, see Table S1 in the supplemental material). The median Cp value of culture-positive samples was 24.6 (IQR, 20.4 to 25.8; range, 17.9 to 30.3). The Cp value was a risk factor for positive CPE (odds ratio, 0.496; 95% confidence interval [CI], 0.329 to 0.747; *P* value, 0.001). Age and coexisting medical conditions were not significantly associated with CPE positivity (*P* values of 0.132 and 0.207, respectively).

10.1128/mSphere.00019-21.1 TABLE S1 Crossing point (Cp) values of PCR tests of asymptomatic SARS-CoV-2 carriers over the course of their infections, with Cp values of culture-positive samples underlined. Download Table S1, DOCX file, 0.02 MB. Copyright © 2021 Murata et al. 2021 Murata et al. https://creativecommons.org/licenses/by/4.0/ This content is distributed under the terms of the Creative Commons Attribution 4.0 International license.

**FIG 2:**
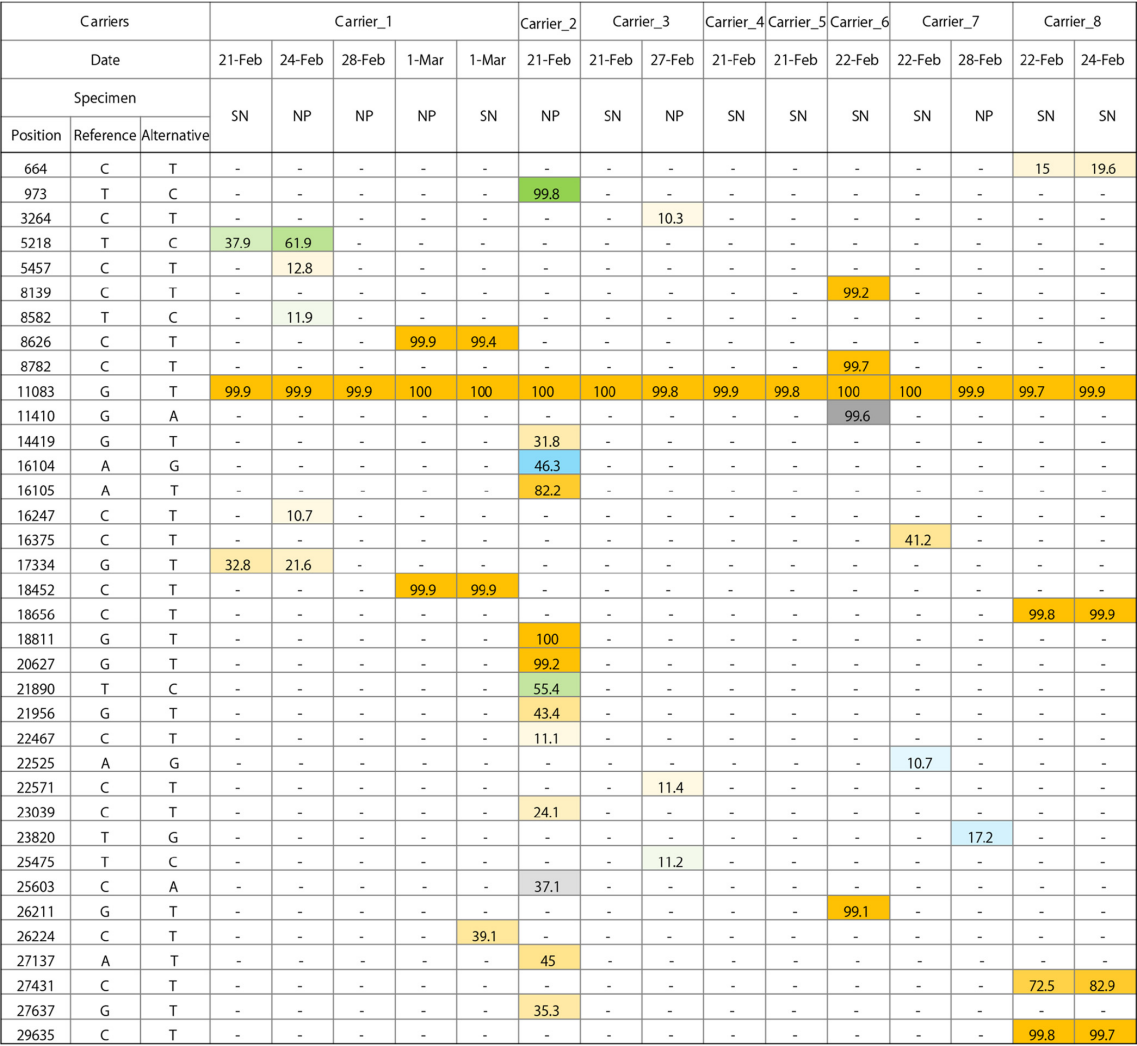
Distribution and allele frequency of SNVs (single nucleotide variants) and intrahost single nucleotide variation (iSNV) identified in specimens obtained from asymptomatic SARS-CoV-2 carriers, compared with the reference genome. Each SNV or iSNV is colored based on the substituted bases (A, gray; G, blue; T, orange; C, green), with color gradients reflecting allele frequencies (in percent). The reference sequence was Wuhan Hu 1 (MN908947.3). SN, supernatant of cell culture; NP, nasopharyngeal swab.

The specimen in which CPE was observed 15 days following the initial positive PCR test was obtained from a 70-year-old Japanese female with a medical history of diabetes mellitus and hypertension, who had prolonged RT-PCR positivity for more than 21 days (Carrier_1 in Fig. 1). In order to explore the virological features, the complete genome sequences of 4 sequential specimens collected from Carrier_1 between 21 February and 1 March, as well as those of 9 specimens obtained from a cabinmate of Carrier_1 (Carrier_2) and 6 others, were obtained and analyzed (Fig. 2). All SARS-CoV-2 strains belonged to clade 19A identified by Nextstrain, and they harbored a single nucleotide mutation at position 11083 (G11083T transversion), leading to a nonsynonymous amino acid substitution (Leu37Phe) in nonstructural protein 6 (nsp6), as previously described from the Diamond Princess outbreak event (9). The consensus sequence obtained from Carrier_1 on 21 and 24 February shared the same intrahost single nucleotide variation (iSNV) at position 5218 with a frequency of 29 to 62% (T5218C transition). While iSNV at position 5218 was not found after 28 February, two novel SNVs emerged in the sample collected on March 1 at positions 8626 (C8626T transition) and 18452 (C18452T transition). The former mutation in the nsp4 gene is synonymous, whereas the latter leads to a nonsynonymous amino acid substitution (Ala138Val) in nsp14, which possesses a 3′-to-5′ exonuclease activity. None of these mutations were found in samples collected from the cabinmate of Carrier_1 and others.

## DISCUSSION

Among 39 asymptomatic SARS-CoV-2 carriers who had more than two positive PCR test results at the hospital, CPE was observed in 9 samples from 7 of the carriers, which were predominantly collected within 7 days after the initial positive PCR testing with Cp values less than 30.3. The rate of CPE positivity was low, likely since the samples from the first positive PCR test, which occurred on the ship and were most likely to yield CPE positivity, were not available and thus were not included in this study.

The viral load (as measured by RT-PCR cycle threshold [*C_T_*] level or Cp level) and duration after symptom onset are known to correlate with SARS-CoV-2 cell infectivity in symptomatic patients; positive viral cultures are mainly observed in samples with *C_T_* values of <34 and those collected within the first week of illness (4, 6, 10–12). In our study, viral load was the leading predictive factor for virus viability among asymptomatic carriers, while age and coexisting medical condition were not. Viable virus was predominantly observed in nasopharyngeal samples obtained within 7 days after the initial positive test, although the time to initial laboratory confirmation varied among individuals due to their persistent asymptomatic status and the ambiguity over the timing of exposure in the setting of a large outbreak event on a cruise ship.

One outlier was a sample obtained from an elderly patient who had prolonged PCR positivity, which showed CPE on day 15 following initial positive PCR testing. Sequencing analysis of the longitudinal nasopharyngeal samples collected from this patient revealed the emergence of two novel SNVs (C8626T transition and C18452T transition) in the sample collected on day 15 after initial PCR confirmation. The C-to-T transitions are the most frequent mutations in SARS-CoV-2, accounting for one-half of sequence variations observed worldwide (13, 14). Considering that these mutations were not identified in virus from the cabinmate of this patient or other passengers, they may have arisen within the host, possibly driven by the human host APOBEC (apolipoprotein B mRNA-editing enzyme, catalytic polypeptide) activity, which deaminates cytosines into uracils (C to U) on single-stranded nucleic acids and confers antiviral intracellular immunity (13, 15). As no mutation was observed in the spike protein sequence, the impact of these mutations on immune evasion is unlikely to be significant.

The strength of this study is that enough nasopharyngeal samples from asymptomatic SARS-CoV-2 carriers were analyzed to demonstrate a link between *in vitro* viral cell infectivity, viral load, and the time from initial laboratory confirmation of SARS-CoV-2 infection. As samples were collected approximately every 48 h from each individual until 2 consecutive negative PCR test results were obtained, the result of this study illustrates the longitudinal kinetics of SARS-CoV-2 infection in asymptomatic carriers. Existing data have suggested that asymptomatic carriers, who are known to have viral loads similar to those of symptomatic patients (16), could serve as a driving force of the virus spreading in the community (1, 2). The results of this study provide additional virologic evidence as to the crucial role of asymptomatic carriers in COVID-19 pandemic, with implications for infection control and public health.

There are several limitations to this study. First, the initial PCR-positive samples which were obtained on the cruise ship were not available for CPE testing. This limitation needs to be acknowledged in interpreting the rate of CPE positivity among asymptomatic carriers, which might be underestimated in the present study as a result. Second, timing of exposure to/infection with SARS-CoV-2 was not ascertained among asymptomatic carriers, making it difficult to estimate how long they had been infected. Third, olfactory and gustatory impairment, which was not recognized as characteristic of COVID-19 at that time, was not routinely checked; thus, those who had isolated olfactory or gustatory symptoms might have been misclassified as asymptomatic.

In conclusion, shedding of viable virus was mainly observed within 7 days following initial laboratory confirmation, except for one person who shed viable virus until day 15, among asymptomatic SARS-CoV-2 carriers who disembarked from the cruise ship Diamond Princess. The viral load was statistically associated with positive viral culture, which was consistent with existing data from symptomatic patients and underscores the role of asymptomatic carriers in driving transmission.

## MATERIALS AND METHODS

### Study population and reverse transcription-PCR testing for SARS-CoV-2 infection.

During the COVID-19 outbreak on the cruise ship Diamond Princess, which occurred in February 2020, asymptomatic passengers and crew members who tested positive for SARS-CoV-2 by screening RT-PCR of nasopharyngeal or throat swabs and their cabinmates who tested negative on the ship were transferred from the cruise ship to on-shore hospitals in Japan for isolation. Asymptomatic status was determined at the time of testing based on the absence of fever (temperature of ≥37.5°C) and clinical symptoms (cough, dyspnea, chest pain, sore throat, and nasal discharge) by physicians and nurses who were mobilized to the ship from quarantine stations, the self-defense forces, and disaster medical assistance teams.

From this cohort, 96 SARS-CoV-2-infected persons who were asymptomatic at the time of testing conducted between 13 and 21 February and their 32 cabinmates who tested negative on the ship were transferred from Diamond Princess to Fujita Health University Okazaki Medical Center, which was slated to open in April 2020, between 19 and 26 February 2020 for continued observation, as previously described (17). The demographics and coexisting medical conditions were extracted from the medical questionnaire obtained upon their arrival to the hospital. Their body temperature, oxygen saturation, and symptoms were monitored at least twice a day, and the nasopharyngeal swabs were collected approximately every 48 h until two consecutive negative PCR test results were obtained. Those who developed signs and symptoms consistent with COVID-19 (i.e., fever of ≥37.5°C, oxygen saturation of ≤93% without supplemental oxygen, or new onset of respiratory symptoms) were transported to nearby acute-care hospitals, since the hospital had not been approved to provide medical care at the time.

All PCR tests were performed at a single government facility under the standard RT-PCR protocol endorsed by the National Institutes of Infectious Diseases. A Cp value of 40 was used as the cutoff value for positivity. During the observation period, 11 of 96 asymptomatically infected persons developed clinical signs and symptoms of COVID-19, making them presymptomatic instead of asymptomatic, and were transferred to other facilities. Meanwhile, eight of 32 cabinmates who had a negative PCR test on the ship had a positive PCR test after arrival to the hospital but remained asymptomatic. By the end of the observation period, after excluding presymptomatic persons and those who were transferred to other facilities due to nonmedical reasons, data on 90 SARS-CoV-2 infected persons who were asymptomatic at the time of testing and remained so throughout, defined as asymptomatic SARS-CoV-2 carriers, were available until the resolution of infection, defined as two consecutive negative PCR tests (17). Among them, the nasopharyngeal swab samples of asymptomatic SARS-CoV-2 carriers who had two or more positive RT-PCR test results at the hospital were analyzed for the presence of viable virus using cell culture, as these samples were more likely than others to yield live virus. This study was approved by the Institutional Review Board of Fujita Health University.

### CPE evaluation.

Nasopharyngeal swab specimens were transported in viral transport medium (BD universal viral transport system), and residual media were stored at −80°C after extraction of RNA for use in RT-PCR testing (17). These frozen media were thawed and processed for culture. The VeroE6/TMPRSS2 cells (Japanese Collection of Research Bioresources Cell Bank, number JCRB1819) were maintained in Dulbecco’s modified Eagle’s medium (DMEM) supplemented with 5% fetal bovine serum (FBS) and penicillin-streptomycin (Sigma-Aldrich). For isolation of SARS-CoV-2, cells were seeded on a 25-cm^2^ cell culture flask (Falcon). Next day, the thawed specimen medium (0.5 ml) was centrifuged at low speed, and the supernatant was mixed with 4.5 ml of isolation medium (DMEM supplemented with 2% FBS, penicillin-streptomycin [Sigma-Aldrich], gentamicin [Sigma-Aldrich], and amphotericin B [Sigma-Aldrich]). The maintenance medium in the flask was then removed, the cells were washed once with isolation medium, and the mixture (5 ml) was added to the flask, followed by incubation at 37°C. We checked whether the cells exhibited CPE, every day for 5 days.

### Whole-genome sequencing and comparative genome sequence analysis of SARS-CoV-2.

SARS-CoV-2 genomic RNA was extracted from culture supernatants and selected nasopharyngeal samples with Cp values less than 34 using a QIAamp Viral RNA minikit (Qiagen). The viral RNA extracted from culture supernatants was subjected to direct MiSeq sequencing as described previously (18, 19). Briefly, a 200-bp fragment library ligated with bar-coded adapters was constructed using an NEBNext Ultra RNA library prep kit for Illumina v1.2 (New England Biolabs) according to the manufacturer’s instructions. The cDNA library was purified using Agencourt AMPure XP magnetic beads (Beckman Coulter). After the quality and quantity of the purified cDNA library had been assessed, nucleotide sequencing was performed on an Illumina MiSeq sequencer (Illumina) using a MiSeq reagent kit v2 (Illumina) to produce 151 paired-end reads. Data analysis of the direct MiSeq sequencing was carried out using CLC Genomics Workbench v8.0.1 (CLC Bio). Contigs were assembled from the obtained sequence reads (trimmed) by *de novo* assembly. To further refine the contigs, the sequence reads were mapped back to the assembled contigs.

In order to obtain longitudinal sequence data, SARS-CoV-2 genomes were amplified from selected nasopharyngeal specimens with Cp values less than 34 from which viable viruses were not isolated according to the PCR protocol of the ARTIC network (https://artic.network/ncov-2019) or using a QIAseq SARS-CoV-2 primer panel (Qiagen). Sequencing libraries of the amplicons were prepared using a QIAseq FX DNA library kit (Qiagen), and the libraries were analyzed with an Illumina MiSeq sequencer, using the MiSeq reagent kit V2, for 300 cycles. Amplicon sequences were mapped to the reference sequence (MN908947.3), and consensus sequences were obtained according to the Utah Department of Health ARTIC/Illumina Bioinformatic Workflow (https://github.com/CDCgov/SARS-CoV-2_Sequencing/tree/master/protocols/BFX-UT_ARTIC_Illumina) with minor modifications to include reads without primers. Consensus sequences obtained from the workflow were confirmed by reading the mapping files (bams) using Integrative Genomics Viewer (IGV) (http://software.broadinstitute.org/software/igv/).

Sequences extracted from each genome were aligned to the reference genome and inspected. Variants with allele frequencies of ≥70% were identified as SNVs (single nucleotide variants). Variant detection was performed using VarScan (20) in addition to manual inspection. An intrahost single nucleotide variation (iSNV) was defined as a variation with an allele frequency of >10% and sequencing depth of ≥1,000. A web application tool (http://giorgilab.dyndns.org/coronapp/) was also used for variant detection and annotation of mutated genes (21).

### Statistical methods.

The data were presented using medians with interquartile ranges (IQRs) and ranges for continuous variables and counts with percentages for binary variables. The impact of Cp values on CPE positivity was assessed by the generalized estimating equations (GEE) approach using an autoregressive (AR-1) correlation matrix to account for correlation among repeated measures. Univariate logistic regression analysis was performed to assess the risk factors for CPE positivity. All *P* values were two-sided. *P* values of <0.05 were considered statistically significant. The statistical analysis was performed using STATA 15.1 and R version 4.0.3.

### Data availability.

The sequences reported in this study are available in the GISAID database and DDBJ Sequence Read Archive and BioSample database. The accession numbers are listed in Table S2 in the supplemental material.

10.1128/mSphere.00019-21.2 TABLE S2 Accession numbers of genome sequences reported in the study. Download Table S2, DOCX file, 0.02 MB. Copyright © 2021 Murata et al. 2021 Murata et al. https://creativecommons.org/licenses/by/4.0/ This content is distributed under the terms of the Creative Commons Attribution 4.0 International license.

## References

[1] Furukawa NW, Brooks JT, Sobel J. 2020. Evidence supporting transmission of severe acute respiratory syndrome coronavirus 2 while presymptomatic or asymptomatic. Emerg Infect Dis 26:e201595. doi:10.3201/eid2607.201595. PMC7323549 32364890

[2] Li R, Pei S, Chen B, Song Y, Zhang T, Yang W, Shaman J. 2020. Substantial undocumented infection facilitates the rapid dissemination of novel coronavirus (SARS-CoV-2). Science 368:489–493. doi:10.1126/science.abb3221. 32179701 PMC7164387

[3] Wölfel R, Corman VM, Guggemos W, Seilmaier M, Zange S, Müller MA, Niemeyer D, Jones TC, Vollmar P, Rothe C, Hoelscher M, Bleicker T, Brünink S, Schneider J, Ehmann R, Zwirglmaier K, Drosten C, Wendtner C. 2020. Virological assessment of hospitalized patients with COVID-2019. Nature 581:465–469. doi:10.1038/s41586-020-2196-x. 32235945

[4] Bullard J, Dust K, Funk D, Strong JE, Alexander D, Garnett L, Boodman C, Bello A, Hedley A, Schiffman Z, Doan K, Bastien N, Li Y, Van Caeseele PG, Poliquin G. 2020. Predicting infectious SARS-CoV-2 from diagnostic samples. Clin Infect Dis 71:2663–2666. doi:10.1093/cid/ciaa638. 32442256 PMC7314198

[5] Arons MM, Hatfield KM, Reddy SC, Kimball A, James A, Jacobs JR, Taylor J, Spicer K, Bardossy AC, Oakley LP, Tanwar S, Dyal JW, Harney J, Chisty Z, Bell JM, Methner M, Paul P, Carlson CM, McLaughlin HP, Thornburg N, Tong S, Tamin A, Tao Y, Uehara A, Harcourt J, Clark S, Brostrom-Smith C, Page LC, Kay M, Lewis J, Montgomery P, Stone ND, Clark TA, Honein MA, Duchin JS, Jernigan JA, Public Health–Seattle and King County and CDC COVID-19 Investigation Team. 2020. Presymptomatic sars-cov-2 infections and transmission in a skilled nursing facility. N Engl J Med 382:2081–2090. doi:10.1056/NEJMoa2008457. 32329971 PMC7200056

[6] van Kampen JJA, van de Vijver DAMC, Fraaij PLA, Haagmans BL, Lamers MM, Okba N, van den Akker JPC, Endeman H, Gommers D, Cornelissen JJ, Hoek RAAS, van der Eerden MM, Hesselink DA, Metselaar HJ, Verbon A, de Steenwinkel JEM, Aron GI, van Gorp ECM, van Boheemen S, van der Eijk AA. 2020. Shedding of infectious virus in hospitalized patients with coronavirus disease 2019 (COVID-19): duration and key determinants. medRxiv doi:10.1101/2020.06.08.20125310. PMC7801729 33431879

[7] Byambasuren O, Cardona M, Bell K, Clark J, McLaws M-L, Glasziou P. 2020. Estimating the extent of asymptomatic COVID-19 and its potential for community transmission: systematic review and meta-analysis. J Assoc Med Microbiol Infect Dis Canada 5:223–234. doi:10.3138/jammi-2020-0030. PMC9602871 36340059

[8] Oran DP, Topol EJ. 2020. Prevalence of asymptomatic SARS-CoV-2 infection: a narrative review. Ann Intern Med 173:362–367. doi:10.7326/M20-3012. 32491919 PMC7281624

[9] Sekizuka T, Itokawa K, Kageyama T, Saito S, Takayama I, Asanuma H, Nao N, Tanaka R, Hashino M, Takahashi T, Kamiya H, Yamagishi T, Kakimoto K, Suzuki M, Hasegawa H, Wakita T, Kuroda M. 2020. Haplotype networks of SARS-CoV-2 infections in the Diamond Princess cruise ship outbreak. Proc Natl Acad Sci U S A 117:20198–20201. doi:10.1073/pnas.2006824117. 32723824 PMC7443927

[10] La Scola B, Le Bideau M, Andreani J, Hoang VT, Grimaldier C, Colson P, Gautret P, Raoult D. 2020. Viral RNA load as determined by cell culture as a management tool for discharge of SARS-CoV-2 patients from infectious disease wards. Eur J Clin Microbiol Infect Dis 39:1059–1061. doi:10.1007/s10096-020-03913-9. 32342252 PMC7185831

[11] Walsh KA, Jordan K, Clyne B, Rohde D, Drummond L, Byrne P, Ahern S, Carty PG, O'Brien KK, O'Murchu E, O'Neill M, Smith SM, Ryan M, Harrington P. 2020. SARS-CoV-2 detection, viral load and infectivity over the course of an infection. J Infect 81:357–371. doi:10.1016/j.jinf.2020.06.067. 32615199 PMC7323671

[12] Cevik M, Tate M, Lloyd O, Maraolo AE, Schafers J, Ho A. 2021. SARS-CoV-2, SARS-CoV, and MERS-CoV viral load dynamics, duration of viral shedding, and infectiousness: a systematic review and meta-analysis. Lancet Microbe 2:e13–e32. doi:10.1016/S2666-5247(20)30172-5. 33521734 PMC7837230

[13] Simmonds P. 2020. Rampant C→U hypermutation in the genomes of sars-cov-2 and other coronaviruses: causes and consequences for their short- and long-term evolutionary trajectories. mSphere 5:e00408-20. doi:10.1128/mSphere.00408-20. 32581081 PMC7316492

[14] Mercatelli D, Giorgi FM. 2020. Geographic and genomic distribution of SARS-CoV-2 mutations. Front Microbiol 11:1800. doi:10.3389/fmicb.2020.01800. 32793182 PMC7387429

[15] Di Giorgio S, Martignano F, Torcia MG, Mattiuz G, Conticello SG. 2020. Evidence for host-dependent RNA editing in the transcriptome of SARS-CoV-2. Sci Adv 6:eabb5813. doi:10.1126/sciadv.abb5813. 32596474 PMC7299625

[16] Lee S, Kim T, Lee E, Lee C, Kim H, Rhee H, Park SY, Son HJ, Yu S, Park JW, Choo EJ, Park S, Loeb M, Kim TH. 2020. Clinical course and molecular viral shedding among asymptomatic and symptomatic patients with sars-cov-2 infection in a community treatment center in the republic of Korea. JAMA Intern Med 180:1447. doi:10.1001/jamainternmed.2020.3862. 32780793 PMC7411944

[17] Sakurai A, Sasaki T, Kato S, Hayashi M, Tsuzuki S-i, Ishihara T, Iwata M, Morise Z, Doi Y. 2020. Natural history of asymptomatic sars-cov-2 infection. N Engl J Med 383:885–886. doi:10.1056/NEJMc2013020. 32530584 PMC7304419

[18] Dennis FE, Fujii Y, Haga K, Damanka S, Lartey B, Agbemabiese CA, Ohta N, Armah GE, Katayama K. 2014. Identification of novel Ghanaian G8P[6] human-bovine reassortant rotavirus strain by next generation sequencing. PLoS One 9:e100699. doi:10.1371/journal.pone.0100699. 24971993 PMC4074113

[19] Komoto S, Adah MI, Ide T, Yoshikawa T, Taniguchi K. 2016. Whole genomic analysis of human and bovine G8P[1] rotavirus strains isolated in Nigeria provides evidence for direct bovine-to-human interspecies transmission. Infect Genet Evol 43:424–433. doi:10.1016/j.meegid.2016.06.023. 27302094

[20] Koboldt DC, Zhang Q, Larson DE, Shen D, McLellan MD, Lin L, Miller CA, Mardis ER, Ding L, Wilson RK. 2012. VarScan 2: somatic mutation and copy number alteration discovery in cancer by exome sequencing. Genome Res 22:568–576. doi:10.1101/gr.129684.111. 22300766 PMC3290792

[21] Mercatelli D, Triboli L, Fornasari E, Ray F, Giorgi FM. 2021. Coronapp: a web application to annotate and monitor SARS-CoV-2 mutations. J Med Virol 93:3238–3245. doi:10.1002/jmv.26678. 33205830 PMC7753722

