# Supplementary material for: Shedding of Viable Virus in Asymptomatic SARS-CoV-2 Carriers
Source: mSphere. 2021 May 19;6(3):e00019-21. doi: 10.1128/mSphere.00019-21 (PMC8265619; doi:10.1128/mSphere.00019-21)
Supplement: TABLE S1 [file msphere.00019-21-st001.docx]

**Supplemental Table 1.** Crossing point (Cp) values of PCR tests of asymptomatic SARS-CoV-2 carriers over the course of their infections, with Cp values of culture positive samples underlined.

| Carriers | Number of days since SARS-CoV-2 infection was diagnosed by a positive PCR test | | | | | | | | | | | | | | | | |
| --- | --- | --- | --- | --- | --- | --- | --- | --- | --- | --- | --- | --- | --- | --- | --- | --- | --- |
|  | 5 | 6 | 7 | 8 | 9 | 10 | 11 | 12 | 13 | 14 | 15 | 16 | 17 | 18 | 19 | 20 | 21 |
| 1 |  |  |  | 36.8 |  |  | 34.3 |  | neg |  | neg |  |  |  |  |  |  |
| 2 |  |  | 32.4 |  | 38.7 |  | neg |  | neg |  |  |  |  |  |  |  |  |
| 3 |  |  | 36.9 |  | 37.0 |  | 37.5 |  | neg |  | neg |  |  |  |  |  |  |
| 4 |  |  | 33.5 |  |  | 37.4 |  | neg |  | neg |  |  |  |  |  |  |  |
| 5 |  |  | 37.8 |  |  | neg |  | 33.6 |  | 35.3 |  | neg |  | neg |  |  |  |
| 6 |  |  | 34.2 |  | 40.0 |  | neg |  | neg |  |  |  |  |  |  |  |  |
| Carrier_3 |  |  | 26.4 |  | 30.3 |  | 38.7 |  | 31.2 |  | neg |  | neg |  |  |  |  |
| 8 |  |  | 36.8 |  | 38.1 |  | neg |  | neg |  |  |  |  |  |  |  |  |
| 9 |  |  | 34.3 |  | 35.2 |  | 40.0 |  | neg |  | 35.4 |  | neg |  | neg |  |  |
| Carrier_4 |  |  | 19.0 |  |  | 28.7 |  | 33.1 |  | 33.9 |  | neg |  | 35.4 |  | neg | neg |
| Carrier_5 |  |  | 21.7 |  | 28.8 |  | 36.8 |  | 37.1 |  | 36.9 |  | neg |  | 37.0 |  | neg |
| 12 |  |  | 37.4 |  | 39.3 |  | neg |  | 35.1 |  | neg |  | neg |  |  |  |  |
| 13 |  | 32.1 |  | 29.2 |  |  | neg |  | neg |  |  |  |  |  |  |  |  |
| 14 |  | 35.7 |  | 32.3 |  | neg |  | neg |  |  |  |  |  |  |  |  |  |
| 15 |  | 32.3 |  | 33.8 |  | 37.9 |  | neg |  | neg |  |  |  |  |  |  |  |
| Carrier_2 |  | 34.3 |  |  | 37.4 |  | neg |  | neg |  |  |  |  |  |  |  |  |
| Carrier_1 |  | 17.9 |  |  | 30.8 |  | neg |  | 33.7 |  | 30.3 |  | 36.7 |  | 39.4 |  |  |
| 18 | 31.3 |  | 36.1 |  | 37.9 |  | neg |  | neg |  |  |  |  |  |  |  |  |
| 19 | 26.0 |  | 40.0 |  | neg |  | neg |  |  |  |  |  |  |  |  |  |  |
| 20 |  |  | 33.7 |  | 35.6 |  | 29.9 |  | 34.0 |  | neg |  | 36.3 |  | neg | neg |  |
| Carrier_6 |  | 24.6 |  | 31.7 |  | 34.6 |  | 36.9 |  | 36.7 |  | neg |  | neg |  |  |  |
| 22 |  | 28.1 |  | 28.6 |  | 31.6 |  | 37.8 |  | 37.9 |  | neg |  | neg |  |  |  |
| 23 |  | 35.8 |  | 36.9 |  | neg |  | neg |  |  |  |  |  |  |  |  |  |
| 24 |  | 33.8 |  | 37.2 |  | neg |  | neg |  |  |  |  |  |  |  |  |  |
| Carrier_7 |  | 25.2 |  | 28.3 |  | 31.8 |  | 32.5 |  | 36.9 |  | neg |  | neg |  |  |  |
| 26 | 26.5 |  | 34.9 |  | 36.3 |  | neg |  | neg |  |  |  |  |  |  |  |  |
| 27 |  | 37.8 |  | 40.0 |  | neg |  | neg |  |  |  |  |  |  |  |  |  |
| 28 |  | 36.1 |  | 37.2 |  | neg |  | neg |  |  |  |  |  |  |  |  |  |
| 29 | 36.5 |  | 37.3 |  | neg |  | neg |  |  |  |  |  |  |  |  |  |  |
| 30 | 23.9 |  | 36.7 |  | neg |  | neg |  |  |  |  |  |  |  |  |  |  |
| 31 | 37.2 |  | 37.3 |  | neg |  | neg |  |  |  |  |  |  |  |  |  |  |
| 32 |  | 29.8 |  | 28.6 |  | neg |  | neg |  |  |  |  |  |  |  |  |  |
| 33 |  |  | 36.7 |  | 36.7 |  | neg |  | 38.2 |  | neg |  | neg |  |  |  |  |
| 34 | 33.4 |  | neg |  | 35.9 |  | neg |  | neg |  |  |  |  |  |  |  |  |
| 35 | 36.8 |  | 33.7 |  | neg |  | neg |  |  |  |  |  |  |  |  |  |  |
| Carrier_8 | 20.4 |  | 24.6 |  | 34.0 |  | 34.1 |  | neg |  | neg |  |  |  |  |  |  |
| 37 |  | 38.1 |  | 37.0 |  | 35.8 |  | 36.2 |  | neg |  | neg |  |  |  |  |  |
| 38 |  | 35.8 |  | neg |  | 36.2 |  | neg |  | neg |  |  |  |  |  |  |  |
| 39 |  | neg |  | 35.7 |  | neg |  | 37.0 |  | neg |  |  | neg |  |  |  |  |
