# Supplementary material for: Shedding of Viable Virus in Asymptomatic SARS-CoV-2 Carriers
Source: mSphere. 2021 May 19;6(3):e00019-21. doi: 10.1128/mSphere.00019-21 (PMC8265619; doi:10.1128/mSphere.00019-21)
Supplement: TABLE S2 [file msphere.00019-21-st002.docx]

**Supplemental Table 2.** Data availability of genome sequences reported in the study.

| Patient | Date of collection | Entry name | GISAID_Accession number | Biosample_Accession number |
| --- | --- | --- | --- | --- |
| Carrier_1 | 2/21/2020 | hCoV-19/Japan/FHU-CS56-0221/2020 | EPI_ISL_728000 | SAMD00268093 |
| Carrier_1 | 2/24/2020 | hCoV-19/Japan/FHU-CS56-0224/2020 | EPI_ISL_728207 | SAMD00268101 |
| Carrier_1 | 2/28/2020 | hCoV-19/Japan/FHU-CS56-0228/2020 | ** | SAMD00268102 |
| Carrier_1 | 3/1/2020 | hCoV-19/Japan/FHU-CS56-0301/2020 | EPI_ISL_728153 | SAMD00268094 |
| Carrier_2 | 2/21/2020 | hCoV-19/Japan/FHU-CS55-0221/2020 | ** | SAMD00268100 |
| Carrier_3 | 2/21/2020 | hCoV-19/Japan/FHU-CS24-0221/2020 | EPI_ISL_718273 | SAMD00268090 |
| Carrier_3 | 2/27/2020 | hCoV-19/Japan/FHU-CS24-0227/2020 | EPI_ISL_728206 | SAMD00268099 |
| Carrier_4 | 2/21/2020 | hCoV-19/Japan/FHU-CS27-0221/2020 | EPI_ISL_727998 | SAMD00268091 |
| Carrier_5 | 2/21/2020 | hCoV-19/Japan/FHU-CS29-0221/2020 | EPI_ISL_727999 | SAMD00268092 |
| Carrier_6 | 2/22/2020 | hCoV-19/Japan/FHU-CS77-0222/2020 | EPI_ISL_728154 | SAMD00268095 |
| Carrier_7 | 2/22/2020 | hCoV-19/Japan/FHU-CS84-0222/2020 | EPI_ISL_728155 | SAMD00268096 |
| Carrier_7 | 2/28/2020 | hCoV-19/Japan/FHU-CS84-0228/2020 | EPI_ISL_728208 | SAMD00268103 |
| Carrier_8 | 2/22/2020 | hCoV-19/Japan/FHU-CS108-0222/2020 | EPI_ISL_728156 | SAMD00268097 |
| Carrier_8 | 2/24/2020 | hCoV-19/Japan/FHU-CS108-0224/2020 | EPI_ISL_728159 | SAMD00268098 |

** Sequences, which were not deposited at GISAID due to frameshifted sequences and gaps, are available in the DDBJ Sequence Read Archive under the accession number DRA011868.
